# Multi-Scale Feature Fusion of Covariance Pooling Networks for Fine-Grained Visual Recognition

**DOI:** 10.3390/s23083970

**Published:** 2023-04-13

**Authors:** Lulu Qian, Tan Yu, Jianyu Yang

**Affiliations:** 1 School of Rail Transportation, Soochow University, Suzhou 215131, China; 2 ByteDance Inc., Moutainview, CA 94042, USA

**Keywords:** fine-grained recognition, covariance pooling, multi-scale feature fusion

## Abstract

Multi-scale feature fusion techniques and covariance pooling have been shown to have positive implications for completing computer vision tasks, including fine-grained image classification. However, existing algorithms that use multi-scale feature fusion techniques for fine-grained classification tend to consider only the first-order information of the features, failing to capture more discriminative features. Likewise, existing fine-grained classification algorithms using covariance pooling tend to focus only on the correlation between feature channels without considering how to better capture the global and local features of the image. Therefore, this paper proposes a multi-scale covariance pooling network (MSCPN) that can capture and better fuse features at different scales to generate more representative features. Experimental results on the CUB200 and MIT indoor67 datasets achieve state-of-the-art performance (CUB200: 94.31% and MIT indoor67: 92.11%).

## 1. Introduction

Image classification is divided into three main categories according to the level of granularity at which the categories are classified: cross-species semantic-level image classification, fine-grained image classification, and instance-level image classification. The fine-grained image classification studied in this paper has been a hot topic in recent years and has a wide range of applications in industry, academia, and everyday life [1,2,3,4]. Fine-grained image classification refers to a more detailed sub-class division based on coarse-grained. Images of different subclasses are often characterized by large intra-class differences and small inter-class differences, making fine-grained image recognition more challenging.

The implementation of image classification is typically divided into three steps: (1) input an array of pixel values for N images, assigning each group of images a corresponding category label for a total of K category labels; (2) use the training set to extract the features of each category and build the model through continuous training and learning; and (3) use the classifier to predict and output the new input image’s classification label. If the predicted label matches the accurate label of the image, the prediction is correct; otherwise, the prediction is incorrect. In the case of fine-grained image classification, the discriminable region is often only a small part of the fine-grained image. Therefore, there are two other aspects to focus on compared to conventional image classification: accurately locating the discriminative key regions and extracting useful features for fine-grained images.

Multi-scale feature fusion techniques and covariance pooling have shown positive performance among the existing fine-grained classification algorithms. However, there are still shortcomings that need to be improved. Existing algorithms that use multi-scale feature fusion for fine-grained classification, such as multi-scale CNNs [5], only focus on the first-order information of an image. The first-order information is often insufficient at distinguishing subtle differences between fine-grained images. Existing fine-grained classification algorithms using covariance pooling, such as BCNN [6] and iSQRT-COV [7], aggregate the local features from the last convolution layer to obtain global representation. Each local feature represents the visual content of a patch of a specific size in the image. Nevertheless, in many cases of fine-grained recognition, the key visual cues may vary in scale across different images. Thus, the local feature representing the patch of a specific size might not effectively describe the comprehensive visual cues.

To solve the above problems, we propose a multi-scale covariance pooling network (MSCPN) that captures the visual content of patches at multiple scales. The MSCPN is comprised of three modules: multi-scale feature extraction, feature fusion, and covariance pooling. In the multi-scale feature extraction module, various pooling methods and kernels are utilized to obtain images at different scales, which are then fed into the baseline network to generate multi-scale feature blocks. In the feature fusion module, the different scale feature maps are combined with the original input feature map using additive fusion to create more informative features. In the covariance pooling module, the fused feature maps are inputted into both BCNN and iSQRT-COV, two typical covariance pooling networks, to capture the second-order information of the image and generate a more representative feature representation. BCNN and iSQRT-COV are used to demonstrate the generalizability of the proposed multi-scale feature fusion technique and to improve the classification accuracy of fine-grained images for better application in practice.

The contributions of this work can be summarized in two aspects:

- We propose a novel fine-grained image classification method based on covariance pooling, which captures second-order information in fine-grained images.
- We propose a multi-scale feature fusion technique, which generates multi-scale feature maps by different pooling methods and different pooling kernel parameters, and then fuses them with the original feature map to obtain better feature representations.

Validation experiments were conducted on two benchmark datasets, including CUB200 [8] and MIT indoor67 [9]. We compared our method with BCNN and iSQRT-COV, and the classification accuracy was improved by 0.9% and 4.6% on CUB200, and 1.5% and 3.3% on MIT indoor67, respectively. In addition, it achieves state-of-the-art results, i.e., 94.31% on CUB200 and 92.11% on MIT indoor67.

The rest of this paper is organized as follows. Section 2 shows the relevant works of this paper. Section 3 describes the details of the proposed multi-scale covariance pooling network. Section 4 presents the classification accuracy and visualization experimental results of this paper’s algorithm on two benchmark datasets. The article is concluded in Section 5.

## 2. Related Work

### 2.1. Fine-Grained Classification Techniques

Fine-grained image classification has emerged as a field since around 2011. Since then, we have divided emergent algorithms into two main categories: traditional algorithms based on feature extraction and deep learning-based algorithms.

#### 2.1.1. Traditional Algorithms Based on Feature Extraction

Traditional algorithms based on feature extraction are based on machine learning techniques. Most of these algorithms extract local features from an image using scale-invariant feature transform (SIFT) [10] or histogram of oriented gradient (HOG) [11]. The feature representation is encoded using models such as the vector of locally aggregated descriptors (VLAD) [12], Fisher vector [13], or bag-of-visual-word (BOVW) [14]. However, the classification results are hardly satisfactory because this type of algorithm is tedious in feature selection, ignores the relationship between different features, requires good manual annotation, and is expensive.

#### 2.1.2. Deep Learning-Based Algorithms

With the rise of deep learning and convolutional neural network technology, researchers have applied it to fine-grained image classification, automatically capturing deep learning features through the network with stronger expressiveness and better classification results. This has greatly facilitated the development of fine-grained image classification algorithms. Fine-grained classification algorithms based on deep learning can be classified into four main categories: (1) CNN-based algorithms; (2) algorithms based on localization-recognition; (3) higher-order coding algorithms based on convolutional features; and (4) algorithms based on network integration.

CNN-based methods: CNNs [15] were first introduced in 1989 by LeCun et al. At that time, CNNs showed superior performance in large-scale visual recognition tasks. Researchers began to consider their application to fine-grained image classification. Subsequently, AlexNet [16] and GoogLeNet [17] were also proposed. In addition to these typical convolutional neural networks, deep convolutional feature extractors include CNN features off-the-shelf [18], InterActive [19], ONE [20], etc. When using these methods, the output of the final fully-connected layer is set to a number of classes for fine-grained image classification. However, with these methods, it is difficult to capture differentiated local details; thus, they are less commonly used today.

Algorithms based on location–recognition: There are two types of algorithms, strongly and weakly supervised, depending on whether additional manual annotation data, such as object annotation boxes, part annotation points, and image category labels, are required. Strongly supervised algorithms often require additional manual annotation information. The parts-based R-CNN [21], proposed by Ning Zhang et al., uses the R-CNN algorithm to detect both the object-level (e.g., dogs) and local areas (head, body, etc.) of fine-grained images. Branson et al. proposed the pose-normalized CNN [22] to perform pose-alignment operations on images, taking into consideration the interference of different bird poses. Shih et al. proposed part localization using multi-proposal consensus [23] to locate key points and regions. Di et al. proposed location alignment classification (Deep LAC) [24] to reduce classification and alignment errors and update localization results adaptively. Weak supervision uses attention mechanisms, clustering, and other methods to automatically obtain distinguishing regions without additional annotation information. They rely solely on classification labels to complete training. Xiao et al. proposed two-level attention [25], it was the first attempt to accomplish weak supervision. Liu et al. proposed the FCN attention model [26], which uses the FCN architecture to select multi-task-driven attention. Bo et al. proposed the diversified visual attention network (DAVN) [27], which uses LSTM to generate different attention regions at additional time steps.

Higher-order coding algorithms based on convolutional features. Higher-order coding methods enhance the representation of features by fusing CNN features to the second order. The bilinear CNN model [6] proposed by Lin et al. uses two CNN networks, one for object part localization and one for object feature extraction. The iSQRT-COV Network [7] proposed by Peihua Li et al. uses an iterative matrix square root normalization algorithm to perform end-to-end training quickly.

Algorithms based on network integration. Network integration, as the name implies, refers to the collocation of multiple neural networks for fine-grained image classification. Network integration can often be used with good results when certain classes are particularly confusing. Ge et al. proposed subset feature-learning networks (SCNs) [28] to cluster similar-looking classes into K subclasses and train K-corresponding specific CNNs. Wang et al. proposed CNN trees [29] to solve multi-classification problems. Ge et al. proposed a mixture of DCNNs [30] to fuse classification results by classification occupation probabilities.

### 2.2. Feature Fusion Techniques

When using convolutional neural networks to extract image features, it is generally considered that the first few layers of the network can extract the lower-level features of the image. As the network deepens, higher-level features of the image can be extracted. Different features have different properties; for example, lower-layer features have higher resolution and contain more positional and detailed information, but they are less semantic and noisier due to having undergone fewer convolutions. Higher-level features have stronger semantic information but have shallower resolution and poorer perception of detail. Therefore, fusing features from different scales is essential for improving image classification. Feature fusion aims to take features extracted from the images and merge them using the complementary nature of the features to create a more discriminative feature than the input features.

Depending on whether feature fusion occurs before or after classifier processing, it can be classified as early fusion (e.g., UNet [31]) or late fusion (e.g., FPN [32]). Early fusion involves fusion at the input layer. Multiple layers of features are fused, and then the predictor is trained on the fused features. Late fusion involves fusion at the prediction layer. Predictions are made on different features, and then these predictions are fused.

Regarding specific techniques, the basic operations of feature fusion can be divided into element-wise addition and concatenation. Element-wise addition adds the values at the corresponding positions of the related channels, with the number of channels remaining unchanged. The number of features in the image remains the same, but the information under each feature increases. Concatenation combines the feature map channels and increases the number of channels. The number of features in the image grows, but the information under each feature remains the same. In addition, feature fusion techniques include feature splicing, outer product expansion between features, skip connections, deconvolution, mask mechanism, gate mechanism [33], etc.

According to the model structure, feature fusion can be divided into a parallel multi-branch structure (e.g., PSPNet [34]) and serially connected structure (e.g., UNet [31]). The model will have multiple branches in a parallel strategy, each with different features. In a serial strategy, the whole model only has one branch. The feature fusion and other components in the network perform operations sequentially.

## 3. Method

**Figure 1:**
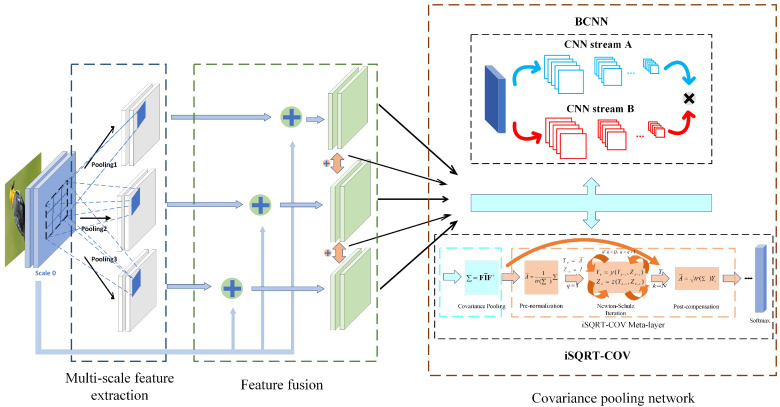
The structure of MSCPN. The feature maps are pooled to generate multiple scales and then fused with the original scales or other generated scales. Finally, they are sent to BCNN and iSQRT-COV. The plus sign in the figure indicates the additive fusion between feature maps. The detailed structures of BCNN and iSQRT-COV are described in Figure 2 and Figure 3.

The framework of our method is shown in Figure 1. The network architecture includes three modules: (1) multi-scale feature extraction, (2) feature fusion, (3) and covariance pooling network. The multi-scale feature extraction module is achieved through different pooling methods and different pooling kernel parameters. The feature fusion module fuses the multi-scale feature maps through additive fusion and sends them to the baseline networks (VGG, ResNet, and DenseNet). The covariance pooling module feeds the fused feature maps into the covariance pooling network for information fusion in the second-order dimension. For a better understanding, the principles of two existing covariance pooling methods (BCNN and iSQRT-COV) are briefly described in Section 3.1. Section 3.2 specifies how the algorithms in this paper are implemented through equations and textual exposition.

### 3.1. Original Bilinear Pooling Network

#### 3.1.1. Bilinear Convolutional Neural Network

**Figure 2:**
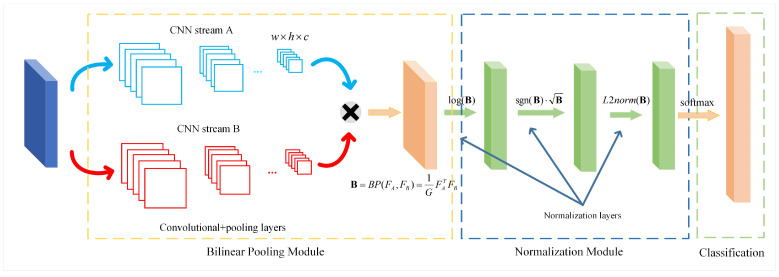
BCNN architecture. The images firstly go through CNNA and CNNB, and use the matrix outer product of each image’s output, whose result then goes through the layers of normalization and classification successively.

The framework of BCNN is illustrated in Figure 2. The BCNN network involves three steps: (1) Feature images are fed into two CNN feature extraction networks, and the feature vectors generated by the two CNN streams are used to compute the outer product of the corresponding position elements, also known as the bilinear pooling process. (2) The feature matrices are normalized through logarithmization, square root, and L2 normalization. (3) The fused features are then fed into the classifier to obtain the classification labels. BCNN can be represented by a quaternion $\mathrm{BP}=(f_{A},f_{B},BP,C)$ for ease of understanding, where $f_{A}$ and $f_{B}$ denote feature extraction functions based on CNNs, BP denotes the bilinear pooling function, and *C* denotes a classification function. Given an input image *I*, the extraction of features using CNN to produce a feature map can be expressed as:

(1) $$F_{A}=f_{A}\left(I\right),F_{B}=f_{B}\left(I\right),$$

where the output image feature map $F_{A}\in R^{M\times c}$, $F_{B}\in R^{N\times c}$. *M* denotes the number of local features in the feature map $F_{A}$. *N* denotes the number of local features in the feature map $F_{B}$. Then, the bilinear pooling function BP obtains the global feature by

(2) $$\begin{array}{c} B=\mathrm{BP}\left(F_{A},F_{B}\right)=\frac{1}{G}F_{A}^{⊤}F_{B}, \end{array}$$

where $B\in R^{M\times N}$ is a bilinear pooling matrix for extracting second-order features. G=M×N denotes the number of features in the feature map after bilinear pooling fusion, i.e., the number of categories to be classified. When the two CNN streams are completely consistent, $f_{A}=f_{B}$ and $F_{A}=F_{B}=F$. Equation (2) is simplified as

(3) $$B=\mathrm{BP}\left(F\right)=\frac{1}{G}F^{⊤}F.$$

At this point, the global eigenmatrix becomes a symmetric semi-positive definite matrix, which is also called second-order pooling (O2P) used in semantic segmentation.

#### 3.1.2. Iterative Matrix Square Root Normalization of the Covariance Pooling Network

**Figure 3:**
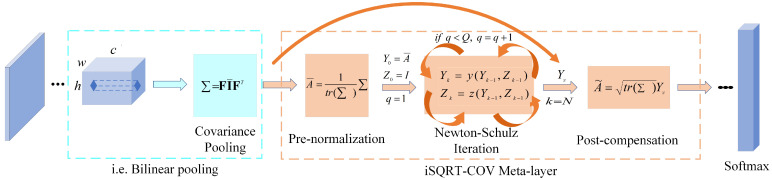
iSQRT-COV architecture.

According to the above description, it is still possible to find a problem with BCNN. BCNN is normalized by elements and does not consider the nonlinear relationship between channels in the feature map, i.e., the manifold structure of covariance matrices. To solve this problem, Li et al. proposed iterative matrix square root normalization of covariance pooling (iSQRT-COV). The structure of iSQRT-COV is shown in Figure 3. The implementation of iSQRT-COV is divided into three steps: (1) Similar to BCNN, the image is fed into the feature extraction network, and the feature matrix is obtained by computing the outer product of the elements at the corresponding positions of its own feature map (i.e., covariance pooling). (2) The feature matrix is fed into the meta layer to calculate the approximate square root. (3) The feature matrix is fed into the classifier to obtain the classification labels. The second step of the meta layer is divided into three parts: pre-normalization, coupling matrix iteration, and post-compensation. iSQRT-COV is not the focus of this paper, so elaboration about the meta layer is not done in this part. The first step of covariance pooling is the same as that of BCNN. Still, BCNN is a two-way feature extraction network followed by outer product positionally. At the same time, iSQRT-COV is a one-way feature extraction network with its feature map for the outer product (positionally). When the two feature extraction networks of BCNN are consistent, the covariance pooling in iSQRT-COV is essentially the same as the bilinear pooling operation in BCNN. To facilitate the description of the algorithm below, we uniformly use the covariance pooling function to refer to the iSQRT-COV and BCNN operations.

### 3.2. Multi-Scale Covariance Pooling Network

The local convolutional features used in covariance pooling networks represent patches of a specific size. However, in many cases, critical visual cues cannot be effectively extracted based on a single-scale network because patches of a particular size may not adequately represent the necessary visual cues for fine-grained recognition.

This paper proposes a multi-scale covariance pooling network (MSCPN) capable of capturing necessary visual cues in different scales and improving the representing power. An overview of the complete structure can be found in Figure 1. The MSCPN implementation is divided into three parts: (1) multi-scale feature extraction (i.e., scaling), (2) feature fusion (i.e., fusing), and (3) covariance pooling. Below, we present the details of the network.

Scaling. We scale the image of a pooling operation *P* to obtain the images in different scales, defined as

(4) $$P_{d,s,p}:I\to \hat{I},$$

where $I\in R^{D\times D}$ denotes the raw image and $\hat{I}\in R^{\hat{D}\times \hat{D}}$ denotes the scaled image. D×D is the size (length×width) of *I* and $\hat{D}\times \hat{D}$ is that of $\hat{I}$. *d* is the size of the pooling kernel, *s* denotes the pooling stride, and *p* denotes the value of padding. They satisfy

(5) $$\hat{D}=\frac{D-d+2*p}{s}+1,d\leq D.$$

The proposed method uses three different pooling kernels ((2, 2, 0), (3, 3, 0), and (3, 3, 1)), and two pooling methods, i.e., average pooling and maximum pooling.

Given an image *I*, we use three scaling functions to obtain the scaled images ${\hat{I}}_{i}$:

(6) $${\hat{I}}_{1}=P_{d_{1},s_{1},p_{1}}^{m_{1}},{\hat{I}}_{2}=P_{d_{2},s_{2},p_{2}}^{m_{2}},{\hat{I}}_{3}=P_{d_{3},s_{3},p_{3}}^{m_{3}},$$

where

(7) $$\begin{array}{c} \left(m_{1},d_{1},s_{1},p_{1}\right)=\left(1,2,2,0\right),\\ \left(m_{2},d_{2},s_{2},p_{2}\right)=\left(2,3,3,0\right),\\ \left(m_{3},d_{3},s_{3},p_{3}\right)=\left(1,3,3,1\right), \end{array}$$

where $m_{i}=1,\left(i=1,2,3\right)$ indicates that average pooling is used, and $m_{i}=2,\left(i=1,2,3\right)$ indicates that maximum pooling is used. $d_{i},s_{i},p_{i}$ correspond to the parameters size, stride, and padding, respectively.

Fusing. We feed the scaled images ${\left\{{\hat{I}}_{i}\right\}}_{i=1}^{3}$ and the original images into the convolution neural network f(·) and generate the feature maps in different scales:

(8) $$F=f\left(I\right),F_{i}=f\left({\hat{I}}_{i}\right),i=1,2,3,$$

(9) $$\hat{F}=\frac{1}{4}\left(F+\sum_{i=1}^{3}F_{i}\right),$$

where $F\in R^{k\times c}$, *k* denotes the number of local features in the feature map F. $F_{i}\in R^{k_{i}\times c}$, where $k_{i}$ is the number of local features in the feature map $F_{i}$. *c* indicates the number of channels in the feature map. $\hat{F}$ denotes the feature map after additive fusion.

In this paper, additive fusion is used, which involves summing the values of two feature map elements at corresponding positions without changing the number of channels in the fused feature map. The use of additive fusion is motivated by its simplicity and computational efficiency, which do not add a significant burden to the network. Furthermore, additive fusion is shown to better capture the spatial location information of the feature map, as suggested by reference [35]. The number of features describing the image remains the same, while the information under each feature is increased, improving the classification performance of fine-grained images.

Covariance pooling. As described in Section 3.1, the fused features are fed into BCNN or iSQRT-COV to obtain the second-order information of the feature maps and perform classification. The covariance pooling process can be expressed as follows:

(10) $$\mathrm{CP}\left(\hat{F}\right)=\frac{1}{G}{\hat{F}}^{⊤}\hat{F},$$

where *G* indicates the number of fine-grained image categories.

After the above description, it is evident that our method in the feature extraction phase is similar to existing multi-scale feature fusion techniques, which use different pooling kernels and methods to obtain feature maps at different scales. However, the difference with existing multi-scale feature fusion lies mainly in the feature fusion stage. The feature fusion part of Figure 1 aggregates information from different scales of feature maps through additive fusion. The covariance pooling network section in Figure 1 innovatively uses covariance pooling (bilinear pooling) to calculate the outer matrix product of the additively fused feature map at each pixel location and then sums it up. In a physical sense, covariance pooling captures the multiplicative interactions of the corresponding spatial locations of the feature map to obtain second-order statistical information on the multi-scale feature map. It makes the feature map information more differentiated and better suited to achieve fine-grained image classification tasks. From this perspective, covariance pooling is a form of feature fusion. However, in this paper, additive fusion and covariance pooling are separated to make the feature fusion process more straightforward for readers to understand.

## 4. Experiment

We validated the MSCPN on two datasets, CUB200 and MIT indoor67. The datasets and implementation details are found in Section 4.1. Ablation studies were conducted to investigate the effectiveness of our proposed method in Section 4.2. The visualization experiments presented in Section 4.3 can more vividly show the advantages of the proposed approach to better represent image features. A comparison with other methods is presented in Section 4.4.

### 4.1. Datasets and Implementation Details

Datasets. Experiments on two classic datasets were conducted for fine-grained image classification, including the CUB200 (Caltech-UCSD Birds) dataset [8] and MIT indoor67 dataset [9]. The CUB200 dataset contains 11,788 images of 200 bird species and the MIT indoor67 dataset contains 15,620 images of 67 indoor categories. The images in these two datasets are divided into two parts: the training set and the test set. These two sets contain nearly the same number of images, with detailed annotations of features and bounding boxes. Birds appear in different poses and viewpoints in the CUB200 dataset. They occupy a small portion of images with cluttered backgrounds, making it more challenging to classify the birds. The indoor scenes are complex and contain multiple objects. Additionally, occlusions and interference between objects are common. Moreover, some indoor scenes (such as corridors) can be discriminated against based on spatial information. Some indoor scenes (such as bookstores) can be determined based on the objects in the background. Hence, the distinguishing features of different scenes make indoor scene recognition challenging. It is worth noting that we only use image labels during training without any annotation of parts and bounding boxes in all of our experiments.

Implementation Details. Our experiments are completed in multi-scale BCNN(MSBCNN) and multi-scale iSQRT-COV(MSiSQRT-COV). Both parts of the experiments are based on the Win10 system and PyTorch framework.

For MSBCNN, the hardware configuration used in the experiments is a server equipped with four NVIDIA GTX 1080ti graphics cards, each with 11G × 4 video memory. Unless stated otherwise, the MSBCNN is implemented through the following general steps. First, each image is resized to 448 × 448 to obtain a finer structure. Next, multi-scale images are generated using various pooling operations. Then, the original and multi-scale images are fed into the feature extraction baseline networks (VGG16, VGG19) respectively, and the resulting feature maps are additively fused. Furthermore, a three-step fine-tuning operation is performed, which involves fine-tuning the FC layers, fine-tuning the entire network, and adding the k-way linear and softmax layers. The FC layers are fine-tuned with a batch size of 64, weight decay of $10^{-8}$, and a learning rate of 1 for 55 epochs. The entire network is fine-tuned end-to-end using back-propagation with a batch size of 64, weight decay of $10^{-5}$, and a learning rate of $10^{-2}$ for 25 epochs. The linear layer is trained using logistic regression. Stochastic gradient descent (SGD) is used to train the loss function of the network.

For MSiSQRT-COV, the hardware configuration of the experiments consists of a server with a TITAN XP graphics card with 12G of video memory. Compared to MSBCNN, the first two steps are the same. The differences are the baseline network used (ResNet50, DenseNet161 for MS-iSQRT-COV) and the subsequent feeding of the feature maps to iSQRT-COV.

Evaluation metric. For quantitative evaluation, classification accuracy serves to measure the effectiveness of our network. It can be obtained from Equation (11).

(11) $$Accuracy=\frac{N_{a}}{N_{t}},$$

where $N_{t}$ denotes the total number of classified images and $N_{a}$ denotes the number of images with correct predictions. The accuracy can reflect the performance of the classification more intuitively, and it is a common classification evaluation metric.

### 4.2. Ablation Study

In order to verify that MSCPN can effectively improve the classification accuracy of fine-grained images, ablation studies and a comparative analysis were conducted.

The effect of different multi-scales. Different pooling kernels were tested, including 2 × 2 with step 2 and no padding, 3 × 3 with step 2 and no padding, and 3 × 3 with step 3 and padding 1. From Table 1, Table 2, Table 3 and Table 4, it can be concluded that the classification performance is better when using the pooling kernel of (3, 2, 0) compared to the other two pooling kernels. In addition, the superposition of different scales was experimented with.

The effect of pooling methods. We present ablation studies on pooling methods: average pooling and max pooling. Pooling results in fewer features and fewer parameters and aims to maintain some invariance (such as rotation, translation, stretching, etc.). Average pooling avoids the error of increasing estimated variance due to the restricted neighborhood size, thus preserving more of the image background information. Max pooling reduces the error in the estimated mean shift caused by errors in the convolution layer parameters, thereby retaining more texture information. Based on Table 1, Table 2 and Table 3, it can be concluded that the average pooling method has better classification performance relatively.

**Figure 4:**
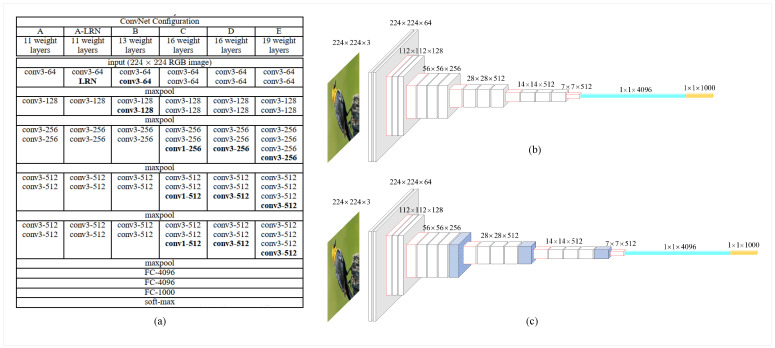
The structure of VGG. (**a**) The structure of VGG, (**b**) The structure of VGG16, (**c**) The structure of VGG19.

The effect of baseline networks. Two feature extraction baseline networks (VGG16 and VGG19) are used for MSBCNN. VGG16 and VGG19 have similar structures and different network depths, as shown in Figure 4. VGG16 consists of thirteen convolutional layers and three fully connected layers. VGG19 consists of sixteen convolutional layers and three fully connected layers. Figure 4a is the overall structure of the VGG network. D (the first row and fifth column) represents VGG16, and the “16 weight layers” directly below it denote that the VGG16 network structure has 16 layers. E (the first line and the sixth column) indicates that VGG19, and the “19 weight layers” directly below it indicate that the VGG19 network structure has 19 layers. Figure 4b,c show the specific network structures of VGG16 and VGG19 in detail, respectively. The three extra network layers of VGG19 over VGG16 are marked in blue shading. From Table 1 and Table 2, it can be concluded that the classification performance of the baseline network VGG19 is higher than that of the baseline network VGG16, further validating that deepening the network depths can improve the performance. ResNet50 and DenseNet161 are used as baseline networks for MSiSQRT-COV. In contrast to ResNet50, each layer of DenseNet161 is explicitly connected to all previous layers within an ensemble region, rather than receiving information only from the latest layer. These connections promote feature reuse, as all other layers can use features from earlier layers. Table 3 shows that the classification performance of the baseline network DenseNet161 is better than that of the baseline network ResNet50.

The effect of upsampling modes. The feature map size changes after multi-scale operations are performed on the image in the initial stage. The same feature map size is required for additively fusing feature maps at different scales. Therefore, an upsampling function is required. The PyTorch framework has several upsampling algorithms: nearest, linear, bilinear, bicubic, and trilinear. In this paper, the experiments tried bilinear and bicubic upsampling algorithms based on MIT indoor67 to validate the MSiSQRT-COV. The results are shown in Table 4. From Table 4, it can be seen that bilinear has better classification results than bicubic.

### 4.3. Visualization

We performed visualization experiments for MSBCNN and MSiSQRT-COV to draw insights into the proposed method.

**Figure 5:**
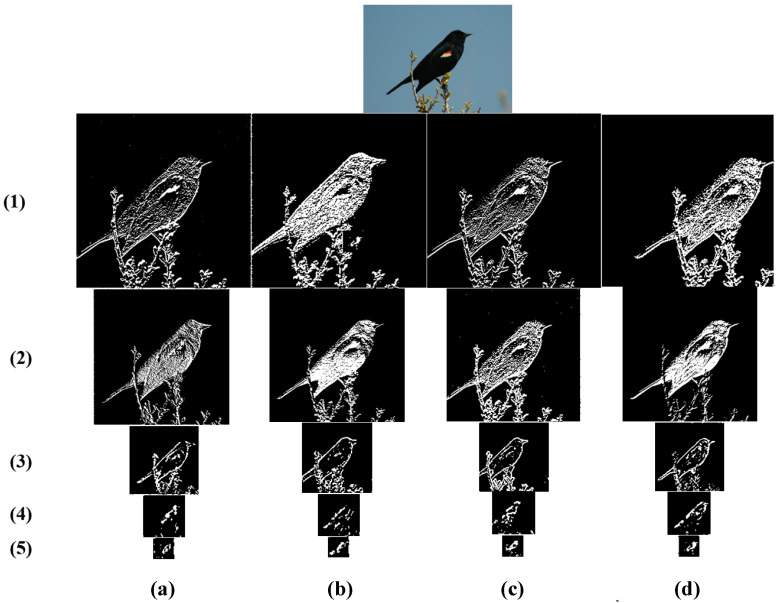
Visualization of MSBCNN based on the baseline VGG16 and VGG19. (**a**) BCNN-CUB200-VGG16, (**b**) MSBCNN -CUB200-VGG16, (**c**) BCNN-CUB200-VGG19, (**d**) MSBCNN-CUB200-VGG19.

For MSBCNN, we randomly selected a test image from the CUB200 dataset and visualized the feature maps with and without multiscale feature fusion based on VGG16 and VGG19, as shown in Figure 5. The first column lists the feature maps of the image generated by BCNN based on VGG16. The second column lists the feature maps of the image generated by MSBCNN based on VGG16. The third column lists the feature maps of the image generated by BCNN based on VGG19. The fourth column lists the feature maps of the image generated by MSBCNN based on VGG19.

As shown in Figure 5, the network structure of VGG16 consists of thirteen convolutional layers and three fully connected layers. The network structure of VGG19 consists of sixteen convolutional layers and three fully connected layers. It is also easy to find that VGG16 and VGG19 have five max pooling layers in the entire network structure. The visualization results in Figure 5 show the feature map outputs of each of the five max pooling layers. The feature maps of these five max pooling layers for visualization were selected because the entire convolutional operation can be divided into five parts from the network structure. Each part of the convolutional operation will result in feature maps of different scales. By comparing these five different scales of feature maps, MSBCNN’s effectiveness in this paper can be better visualized.

The bright white parts in the visualizations are the regions to which the network especially pays attention. High-level to low-level feature maps are displayed from the first row to the fifth row. Low-level feature maps tend to extract textures and details. In contrast, high-level function maps tend to extract the most representative contours, forms, and characteristics. The higher the levels, the more representative the extracted features.

Comparing (a) with (b) (or (c) with (d)) in Figure 5, on the low-level feature maps, the feature maps generated by MSBCNN are brighter than that generated by BCNN in detail, and the texture is more pronounced. For example, in row 1(b) and row 2(b), the colorful feathers on the bird’s wings are brighter, and the texture of the bird’s feathers is more detailed and hierarchical. The outline of the target in the high-level feature maps generated by our proposed method is more complete. Specifically, the bird’s outline in row 3(a) is less complete, while the bird’s outline in row 3(b) is relatively complete. Moreover, in the high-level feature maps, the features represented by the bright white parts are more representative. Specifically, in row 5(b), the details of the beak and most of the bird’s body are visible.

Comparing (b) with (d) (or (a) with (c)), when the baseline network is VGG19, the texture of the low-level feature map is more hierarchical and more accurate in capturing the bird’s features (e.g., in row 1(d) and row 2(d), the part of the bird’s feathers is brighter and whiter). Furthermore, the high-level feature map has more apparent contours. It better captures the most representative features (e.g., in row 3(d), the bird’s contours are more complete. In row 4(d) and row 5(d), the features of the bird’s body and beak are captured more thoroughly). Thus, the classification performance is better when the baseline network is VGG19.

**Figure 6:**
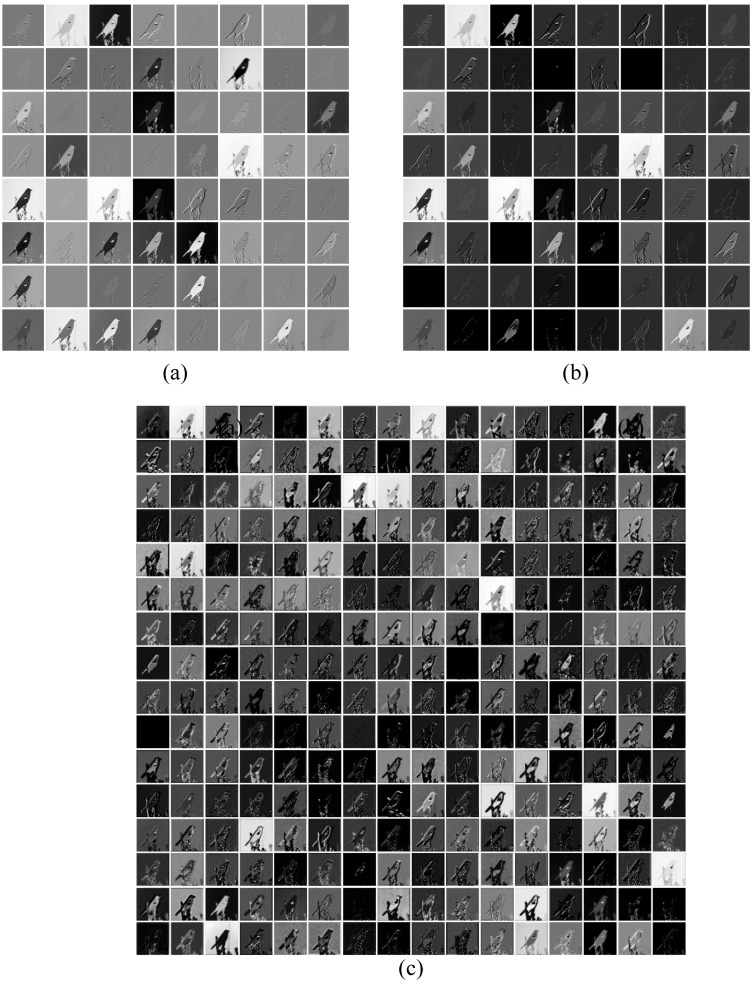
Visualization of iSQRT-COV based on the baseline ResNet50. (**a**) Visualization of the first layer of the network, (**b**) Visualization of the third layer of the network, (**c**) Visualization of the fifth layer of the network.

**Figure 7:**
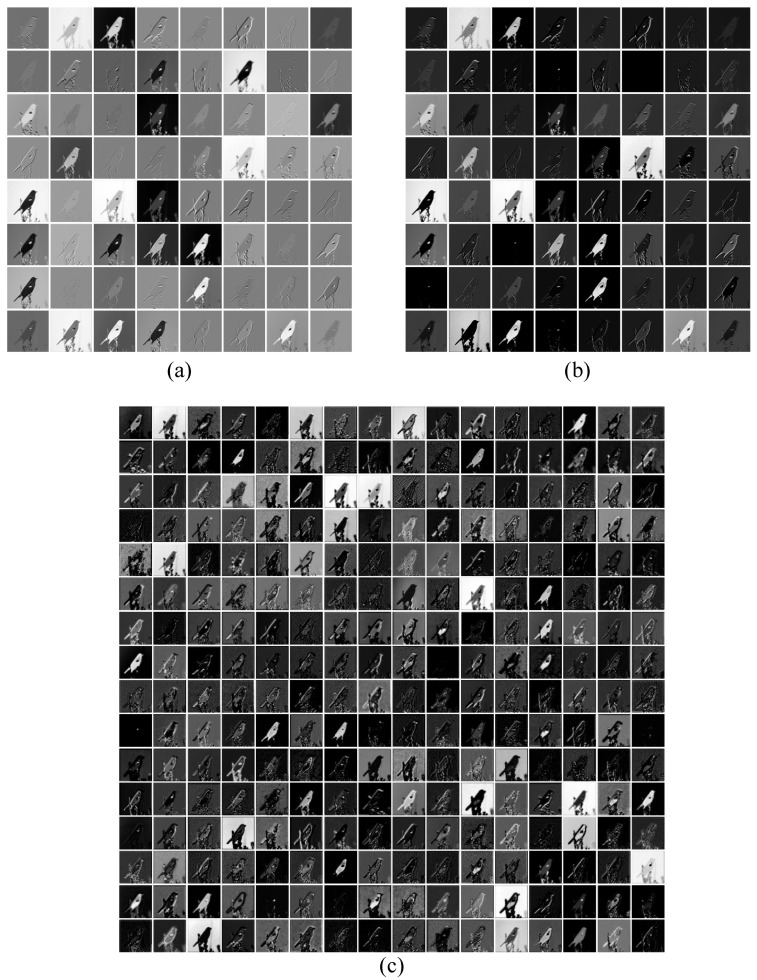
Visualization of MSiSQRT-COV based on the baseline ResNet50. (**a**) Visualization of the first layer of the network, (**b**) Visualization of the third layer of the network, (**c**) Visualization of the fifth layer of the network.

Similarly, for MSiSQRT-COV, a test image from the CUB200 dataset is randomly selected and fed into the network to visualize the feature maps of each layer, as shown in Figure 6 and Figure 7. Figure 6 and Figure 7 show the output feature maps of iSQRT-COV and MSiSQRT-COV based on three network layers of ResNet50 and DenseNet161. Each small image represents the feature map under the corresponding network layer under different channels. Comparing (a), (b), and (c) in Figure 6 and Figure 7, we can find that the contours, lines, and details of the feature maps processed by multi-scale feature fusion are clearer than those of the original scale at the same position in the network layer.

### 4.4. Comparison to Other Methods

#### 4.4.1. Comparison to Original Covariance Pooling Methods

Table 5 compares BCNN and MSBCNN on CUB200 and MIT indoor67. Compared with BCNN, the accuracy of the two datasets is improved by 0.9% and 1.5%, respectively. It can be concluded that our proposed method is indeed superior to BCNN in classification accuracy, and the improvement on MIT indoor67 is more obvious. At the same time, Table 5 shows the comparison between iSQRT-COV and MSiSQRT-COV on CUB200 and MIT indoor67. Compared with iSQRT-COV, the accuracy of both datasets is improved by 4.6% and 3.3%, respectively. It can be summarized that the proposed approach is indeed superior to iSQRT-COV in fine-grained recognition, and the improvement of CUB200 is more obvious.

#### 4.4.2. Comparison with the Existing Methods

Table 6 shows the performance of our method on CUB200 and MIT indoor 67 and its comparison with other methods. Our method achieves bird classification accuracy of 94.3% and indoor scene recognition accuracy of 92.1%. In summary, MSCPN is markedly superior to the compared existing methods.

## 5. Conclusions

In this work, we proposed a new multi-scale feature fusion technique (MSCPN), which captures the visual content of patches in multiple scales and fuses their bilinear features based on covariance pooling networks. Unlike previous work, the proposed network considers both multi-scale and second-order information in the features. Experiments on birds and scene recognition verify the effectiveness of the novel work. In the future, we will conduct extensive research in two directions, i.e., we will investigate whether fusing inter-layer features [58,59] can effectively obtain feature representations at multiple scales and we will explore how to merge other effective methods to improve multi-scale covariance pooling networks to learn better fine-grained representations.

## Figures and Tables

**Table 1:** Classification accuracy on CUB200 for MSBCNN.

Scale	Multi-Scale Approach	Baseline	Accuracy		Baseline	Accuracy	
			FC	ALL		FC	ALL
(1)	Avg pooling (2,2,0)	VGG16	78.50	84.96	VGG19	77.79	84.94
(2)	Max pooling (2,2,0)		78.24	84.57		77.55	84.80
(3)	Avg pooling (3,2,0)		79.04	84.67		78.05	84.79
(4)	Max pooling (3,2,0)		78.29	84.96		77.10	85.13
(5)	Avg pooling (3,3,1)		78.89	84.74		76.27	84.21
(6)	Max pooling (3,3,1)		77.96	84.17		74.65	83.87
(7)	(1)+(1)	VGG16	78.79	84.56	VGG19	77.79	84.67
(8)	(2)+(2)		78.19	84.56		77.55	85.01
(9)	(1)+(2)		78.19	84.39		77.55	84.86
(10)	(1)+(4)		77.93	84.37		77.10	84.39
(11)	(1)+(5)		78.79	85.12		77.79	84.96
(12)	(4)+(5)		78.29	84.94		77.10	84.99
(13)	(1)+(4)+(5)		78.31	84.99		77.12	85.13

The first column lists the number of different scales. The second column lists the fusion strategies at different scales. The third and sixth columns list the baseline networks used in the experiments. The fourth and seventh columns list the accuracy after fine-tuning only FC layers, and the fifth and eighth columns list the accuracy after fine-tuning all layers.

**Table 2:** Classification accuracy on MIT indoor67 for MSBCNN.

Scale	Multi-Scale Approach	Baseline	Accuracy		Baseline	Accuracy	
			FC	ALL		FC	ALL
(1)	Avg pooling (2,2,0)	VGG16	77.64	79.69	VGG19	76.86	79.94
(2)	Max pooling (2,2,0)		78.20	79.97		76.90	80.28
(3)	Avg pooling (3,2,0)		77.43	80.05		77.93	81.00
(4)	Max pooling (3,2,0)		77.49	80.31		76.31	80.14
(5)	Avg pooling (3,3,1)		77.28	80.04		76.72	79.51
(6)	Max pooling (3,3,1)		77.25	80.14		77.43	80.13
(7)	(1)+(1)	VGG16	78.79	80.05	VGG19	77.86	80.77
(8)	(2)+(2)		77.59	80.02		76.55	79.96
(9)	(1)+(2)		77.57	80.24		76.87	80.18
(10)	(1)+(4)		77.46	80.08		76.78	80.15
(11)	(1)+(5)		77.35	80.34		77.77	80.64
(12)	(4)+(5)		77.52	80.11		76.57	79.97
(13)	(1)+(4)+(5)		76.32	79.59		76.64	80.07

**Table 3:** Classification accuracy on CUB200 and MIT indoor67 for MSiSQRT-COV.

Scale	Multi-Scale Approach	Baseline	Accuracy		Baseline	Accuracy	
			CUB200	MIT Indoor67		CUB200	MIT Indoor67
(1)	Avg pooling (2,2,0)	ResNet50	91.18	87.62	DenseNet161	94.04	91.39
(2)	Max pooling (2,2,0)		91.02	88.20		93.78	91.48
(3)	Avg pooling (3,2,0)		91.36	88.56		94.14	92.10
(4)	Max pooling (3,2,0)		90.96	87.28		93.69	90.48
(5)	Avg pooling (3,3,1)		90.97	88.70		93.48	91.22
(6)	Max pooling (3,3,1)		90.72	87.70		93.03	91.77
(7)	(1)+(1)	ResNet50	91.43	87.82	DenseNet161	94.16	91.17
(8)	(2)+(2)		91.04	87.94		93.88	89.42
(9)	(1)+(2)		91.28	87.57		93.92	91.52
(10)	(1)+(3)		91.14	86.91		93.24	91.27
(11)	(1)+(5)		91.31	87.39		94.29	90.14
(12)	(3)+(5)		91.31	87.37		93.44	91.44
(13)	(1)+(3)+(5)		91.61	87.55		93.41	92.13

The first column lists the number of different scales. The second column lists different strategies at different scales. The third and sixth columns list the baseline network used in the experiment. The fourth, fifth, seventh, and eighth columns list the accuracy on CUB200 and MIT indoor67, respectively.

**Table 4:** Classification accuracy under different upsampling modes.

Model	Scale	Multi-Scale Approach	Baseline	Mode	
				Bicubic	Bilinear
MSiSQRT-COV	(1)	Avg pooling (2,2,0)	ResNet50	87.49	87.62
	(2)	Max pooling (2,2,0)		88.10	88.20
	(3)	Avg pooling (3,2,0)		87.34	88.56
	(4)	Max pooling (3,2,0)		86.86	87.28
	(5)	Avg pooling (3,3,1)		87.34	88.90
	(6)	Max pooling (3,3,1)		87.67	87.70

The experiments tried bilinear and bicubic upsampling algorithms based on MIT indoor67 to validate the MSiSQRT-COV. The first column indicates that the experimentally validated model is MSiSQRT-COV. The second column lists the number of different scales. The third column lists different strategies at different scales. The fourth column indicates that the baseline network used in this ablation experiment is ResNet50. The fifth and sixth columns enumerate the classification accuracy in bicubic and bilinear modes.

**Table 5:** Comparison between BCNN and iSQRT-COV. The first column lists different methods of fine-grained classification. The second column shows the baseline. The third and fourth columns list the classification results corresponding to each dataset.

Method	Baseline	CUB200	MIT Indoor67
		Accuracy	Accuracy
BCNN	VGG16	84.1	78.9
	VGG19	84.3	79.5
Multi-scale BCNN	VGG16	85.0	80.3
	VGG19	85.1	81.0
iSQRT-COV	ResNet50	87.0	86.4
	DenseNet161	93.2	90.6
Multi-scale iSQRT-COV	ResNet50	91.6	88.7
	DenseNet161	94.3	92.1

**Table 6:** Comparisons with state-of-the-art methods.

CUB200				MIT Indoor67			
Method	Baseline	Accuracy	Year	Method	Baseline	Accuracy	Year
SENet [36]	SENet-154	80.8	2018	MS-CNNs [5]	VGG16	86.04	2016
CutMix [37]	ResNet50	83.6	2019	HoAS [38]	AlexNet	88.2	2018
CPM [39]	GoogLeNet	87.7	2019	SENet [36]	ResNet101	89.1	2018
MS feature fusion [40]	ResNet101	85.7	2020	WS-AM [41]	VGG11	85.7	2019
API-NET [42]	DenseNet-161	87.7	2020	M2M BiLSTM [43]	ResNet50	88.3	2019
CIN [44]	ResNet-101	88.1	2020	Two-class SVM-fuzzy [45]	ResNet50	73.6	2020
ViT [46]	ViT-B-16	90.6	2021	FOSNet [47]	ResNet50	90.3	2020
WS-DAN [48]	ResNet50	84.91	2021	MMD [49]	UperNet 50	87.10	2020
SnapMix [50]	ResNet-101	89.6	2021	DPP [51]	ResNet101	90.8	2021
RCL [52]	ResNet50	89.8	2022	SMILE [53]	ResNet50	85.1	2021
CMSEA [54]	EfficientNetV2-S	90.6	2022	MRNet [55]	ResNet50	88.1	2022
TPSKG [56]	ViT-B-16	91.3	2022	PlacesNet+ObjectNet [57]	MR-CNNs	90.3	2022
Ours	DenseNet161	94.3	2022	Ours	DenseNet161	92.1	2022
	ResNet50	91.6	2022		ResNet50	88.7	2022

## Data Availability

Data sharing not applicable. No new data were created or analyzed in this study. Data sharing is not applicable to this article.
